# Liquid biopsy in mice bearing colorectal carcinoma xenografts: gateways regulating the levels of circulating tumor DNA (ctDNA) and miRNA (ctmiRNA)

**DOI:** 10.1186/s13046-018-0788-1

**Published:** 2018-06-26

**Authors:** Jessica Gasparello, Matteo Allegretti, Elisa Tremante, Enrica Fabbri, Carla Azzurra Amoreo, Paolo Romania, Elisa Melucci, Katia Messana, Monica Borgatti, Patrizio Giacomini, Roberto Gambari, Alessia Finotti

**Affiliations:** 1 Department of Life Sciences and Biotechnology, Biochemistry and Molecular Biology Section, Ferrara University, Via Fossato di Mortara 74, 44121 Ferrara, Italy; 2 Oncogenomics and Epigenetics, IRCSS Regina Elena National Cancer Institute, Via E. Chianesi 53, 00144 Rome, Italy; 3 Pathology, IRCSS Regina Elena National Cancer Institute, Rome, Italy

**Keywords:** Colorectal carcinoma, Liquid biopsy, ctDNA, ctmiRNA, Tumor xenotransplants

## Abstract

**Background:**

Circulating tumor DNA (ctDNA) and miRNA (ctmiRNA) are promising biomarkers for early tumor diagnosis, prognosis and monitoring, and to predict therapeutic response. However, a clear understanding of the fine control on their circulating levels is still lacking.

**Methods:**

Three human colorectal carcinoma cell lines were grown in culture and as tumor xenograft models in nude mice. Chip-based and droplet digital PCR platforms were used to systematically and quantitatively assess the levels of DNAs and miRNAs released into the culture supernatants and mouse blood plasma.

**Results:**

Strikingly, mutated DNAs from the same (KRAS) and different (PIK3CA and FBWX7) genomic loci were differentially detected in culture supernatants and blood, with LS174T releasing 25 to 60 times less DNA in culture, but giving rise to 7 to 8 times more DNA in blood than LoVo cells. Greater LS174T ctDNA accumulation occurred in spite of similar CD31 immunostaining (micro-vascularization) and lesser proliferation and tissue necrosis as compared to LoVo. As to the three selected miRNAs (miR-221, miR-222 and miR-141), all of them were constitutively present in the plasma of tumor-free mice. Micro-RNA miR-141 was released into HT-29 cell supernatants 10 and 6.5 times less abundantly with respect to LoVo and LS174T, respectively; on the contrary, release of miR-141 in blood of HT-29 xenografted mice was found similar to that observed in LoVo and LS174T mice.

**Conclusions:**

Taken together, our results support the existence of multiple, finely tuned (non-housekeeping) control gateways that selectively regulate the release/accumulation of distinct ctDNA and miRNA species in culture and tumor xenograft models. Different xenografts (proxies of different patients) considerably differ in gateway usage, adding several layers of complexity to the well-known idea of molecular heterogeneity. We predict that even high tissue representation of mutated DNA and miRNA may result in insufficient diagnostic analyte representation in blood. In this respect, our data show that careful modeling in mice may considerably help to alleviate complexity, for instance by pre-screening for the most abundant circulating analytes in enlarged sets of tumor xenografts.

**Electronic supplementary material:**

The online version of this article (10.1186/s13046-018-0788-1) contains supplementary material, which is available to authorized users.

## Background

Soluble biomarkers in body fluids, particularly those circulating in the bloodstream, provide key diagnostic information in several human diseases, including cancer. The carcinoembryonic antigen (CEA), for instance, has been used for decades to diagnose and monitor cancer progression, since elevated levels are associated with cancer spreading. However, as for many other ‘first generation’ biomarkers, circulating levels of this analyte are variable in healthy individuals and often overlap in unaffected and diseased populations, detracting from their general applicability [1–3].

In the last few years, the introduction of precision diagnostics has revolutionized the entire biomarker field. Unlike conventional markers, ‘precision’ biomarkers such as circulating tumor DNA (ctDNA) and miRNA (ctmiRNA) reveal genetic and epigenetic aberrations that directly drive cancer [4]. In addition, unique mutations of ctDNAs are virtually cancer-specific, and ctmiRNA signatures pinpoint specific biological features. Indeed, every cancer patient is believed to host a unique set of tumor aberrations (e.g. DNA mutations and/or overexpressed miRNAs), each of which can be detected in the blood and used as a surrogate or ‘index’ biomarker for cancer diagnosis, prognosis, therapeutic assignment, and follow-up [4].

Detection of ctDNA/ctmiRNAs in body fluids, usually referred to as liquid biopsy, has demonstrated great potential. However, this approach also suffers from some limitations, both theoretical and technical [5, 6]. Extreme personalization may be challenging. The biological processes underpinning release of index aberrations are complex, highly dependent on the analyte being considered, and may differ in different patients. RAS mutations, for instance, are often subclonal, preventing their consistent appreciation in a liquid biopsy format [7]. Therefore, capturing the mutational complexity of cancer by liquid biopsy will require the combination of multiple index aberration, ideally from different classes of analytes, into a single multi-marker assay.

In light of this, it is mandatory to address the variables determining the availability of circulating analytes and design optimized multi-marker panels, but this may prove extremely challenging and ethically questionable if one exclusively relies on human specimens. Additionally, in vitro spiking experiments with known amounts of DNAs/miRNAs may fail to resolve the mechanisms of analyte formation, release in the extracellular spaces, export into the bloodstream, and eventually persistence in a soluble, detectable form. Therefore, controlled animal models are required to optimize liquid biopsy approaches, and spare ethically sensitive clinical material from cancer patients.

In this study, we take advantage of chip-based and droplet digital PCR platforms and enumerate ctDNAs and miRNAs released from three human colorectal carcinoma (CRC) cell lines with different KRAS genotypes. We systematically compare levels of mutations from the same (KRAS) and different (PIK3CA and FBWX7) genomic loci and miRNAs (namely miR-221, miR-222, and miR-141) in cell extracts and culture supernatants on the one hand, and the corresponding mouse tumor xenografts and matched blood samples from these mice on the other. This simple and informative experimental model reveals the existence of crucial gateways that are likely to determine biological fates and clinical usefulness of single ctDNA and ctmiRNA species in individual patients.

## Methods

### Cell lines and supernatants

Human CRC cells (HT-29, LoVo and LS174T) were purchased from the American Type Culture Collection (ATCC). HT-29 cells were derived from a KRAS-WT, differentiated colorectal adenocarcinoma. LoVo cells (originally described as Dukes’ type C, grade IV) and LS174T cells (Dukes’ type B) harbor a heterozygous KRAS c.38G > A mutation (G13D) and a heterozygous KRAS c.35G > A mutation (G12D), respectively. Somatic mutations and their Variant Allelic Frequencies (VAFs) were confirmed by interrogating the *Cancer Cell Line Encyclopedia* (*https://portals.broadinstitute.org/ccle**).* Cell identity was confirmed by HLA genotyping, as described [8]. Cells were grown in RPMI 1640, 10% fetal bovine serum, 1% l-glutamine and 1% penicillin/streptomycin (all from Euroclone, Milan, Italy) in a humidified atmosphere containing 5% CO_2_. To permit the accumulation of DNA and miRNAs in culture supernatants, cells were grown for 72 h to 70% confluency and manually counted by the Trypan Blue exclusion method. Cells were harvested and either pelleted and snap-frozen (for miRNA extraction, see below), or Formalin Fixed and Paraffin Embedded (FFPE), as routinely done for histological specimens (for DNA extraction, see below). Spent medium was then collected, spun twice at 4 °C (2000×g for 20 min and 16,000×g for 10 min), and immediately stored at − 80 °C in aliquots.

### Tumor xenografts and plasma preparation

Animal studies were performed according to Directive 2010/63/EU and Italian Decree Law 26/2014. They were approved by the EU Research Executive Agency (REA), the Intramural Regina Elena Board for Animal Welfare, and the Italian Ministry of Health (prot n. 700–2015-PR, dated July 17th, 2015). Tumor xenotransplants were established by inoculating 3 × 10^6^ cells from the HT-29, LS174T and LoVo cell lines in the flank of 4-month old Nu/CD1 mice (Charles River Laboratories, Italy). Xenotransplants were allowed to grow to two different sizes (300 and 1000 mm^3^, 6 mice per group for each of the three cell lines). Additional independent experiments were carried out on smaller cohorts of mice and individual animals. Tumors were taken at sacrifice along with blood, and were divided into two fragments: one half was snap-frozen in liquid nitrogen, the other half was FFPE-processed exactly as routinely done for human diagnostic histopathology. Frozen tissues were used as the source of miRNAs, FFPE samples were used as the source of genomic DNA, for morphological evaluation (hematoxylin/eosin stain), and for immunohistochemistry (see Additional file 1). Blood was collected in 6 mL BD Vacutainer K_2_E tubes (BD, #368857) and centrifuged within 1 h at 2,000×g for 20 min at 4 °C. Plasma was recovered and further centrifuged at 16,000×g for 10 min at 4 °C to remove cell debris, and stored at − 80 °C until extraction.

### DNA extraction

DNA was obtained according to the manufacturer’s (Qiagen, Hilden, Germany) instructions from four different sources. Genomic DNA was extracted by the QIAamp® DNA FFPE Tissue Kit from FFPE inclusion slices of either (a) cell pellets of cultured cells or (b) tumor xenotransplants. ctDNA was extracted with the QIAamp® CNA kit from either (c) 2 mL of culture supernatants or from (d) 2 mL of mouse plasma. DNAs from (c) and (d) were eluted in a final volume of 30 μL, and stored at − 20 °C. All DNAs were quantified with the Qubit dsDNA assay kit (LifeTechnologies, Carlsbad, CA, USA) prior to dPCR analysis.

### miRNA extraction

RNA was extracted from frozen cell (5 × 10^5^) pellets by the TRI-Reagent (Sigma-Aldrich, St.Louis, MO, USA) and from cell culture supernatants according to Turchinovich et al. [9] with minor modifications. Briefly, 400 μL of culture supernatants were lysed in 1.2 ml of TRIzol LS (Thermo Fisher Scientific, Waltham, MA, USA) in the presence of 12 μg of carrier glycogen (Thermo Fisher Scientific) and 400 attomoles of *C. elegans* miR-39-3p (cel-miR-39-3p) (Thermo Fisher Scientific) to control miRNA recovery efficiency. Following phase-partitioning by nuclease-free chloroform (Sigma-Aldrich), miRNAs were purified using miRNeasy Serum/Plasma columns (Qiagen). RNA was eluted in 18 μL of nuclease-free water (Sigma-Aldrich). The snap-frozen tumor fragment was homogenized using an IKA T10 Basic Ultraturrax (IKA Werke, Staufen, DE) in TRI-Reagent at maximum speed for 1 min and then processed as above. For the analysis of ctmiRNA, blood plasma (150 μL) was treated to disrupt exosomes and denaturate miRNA-binding proteins in 5 volumes of QIAzol Lysis Reagent (Qiagen). After the addition of 400 amoles of cel-miR-39-3p, total RNA was purified with the miRNeasy Serum/Plasma Kit (Qiagen) in a final volume of 18 μL. All RNAs were stored at − 80 °C until use.

### Digital PCR on genomic DNA, supernatants, mouse tissues and ctDNA

DNA samples were dispensed in the chip-based QuantStudio™ 3D Digital PCR System (Thermo Fisher Scientific), and amplified using selected TaqMan™ SNP Genotyping Assays for human KRAS mutations (Table 1). dPCR reactions were set up in a final volume of 15 μL, containing 7.5 μL of 2× QuantStudio™ 3D Digital PCR Master Mix, 0.75 μL of TaqMan® SNP Genotyping assays (Life Technologies), and different DNA templates, each adjusted in 6.75 μL water, as follows. Genomic DNAs from cells and tumor tissues were 50 ng; DNAs from culture supernatants were 6.75 μL equivalent to 450 μl of supernatant; ctDNAs were 6.75 μL equivalent to 45 μl plasma. Thermal cycling was as follows: 10 min at 96.0 °C, 39 cycles at 56.0 °C for 2 min, 30 s at 98.0 °C, and a final elongation step of 2 min at 60 °C. Copy numbers were calculated assuming that a human haploid genome DNA corresponds to 3.3 pg DNA.

**Table 1:** TaqMan assays (primer and probe sets) employed in dPCR, RT-ddPCR and RT-experiments

Assay (primers and probe)	Assay code^a^	Employed technology
KRAS p. G12D	KRAS_521, ID AH6R5PI^b^	dPCR
KRAS p. G13D	KRAS_ 532, ID AHD2BW0^b^	dPCR
PIK3CA p. H1047R	PIK3CA_775, ID AHPAVCD^b^	dPCR
FBXW7 p. R505C	custom assay, ID ANZTGJU^b^	dPCR
hsa-miR-141-3p	000463^c^	RT-ddPCR, RT-qPCR
hsa-miR-221-3p	000524^c^	RT-ddPCR, RT-qPCR
hsa-miR-222-3p	002276^c^	RT-ddPCR, RT-qPCR
cel-miR-39-3p	000200^c^	RT-ddPCR, RT-qPCR

^a^All assays are provided from Thermo Fisher Scientific. ^b^This assay was used for dPCR on genomic DNA and ctDNA. ^c^This assay was used for reverse transcription and amplification of microRNAs in ddPCR and quantitative PCR

### Reverse transcription droplet digital PCR (RT-ddPCR) assays for microRNA expression analysis

Levels of miR-141, miR-221 and miR-222 were assessed by Reverse Transcription droplet digital PCR (RT-ddPCR). 300 ng of total RNA (from cells and tissues) or 3 μL of purified miRNAs (corresponding to 65 μL of supernatants and 25 μL of plasma) were reverse transcribed using the TaqMan™ miRNA Reverse Transcription Kit (Thermo Fisher Scientific) according to the manufacturer’s manual, in a final reaction volume of 20 μl. 1.3 μL of cDNA, undiluted (for supernatants and plasma) or diluted 1:100 (for cells and tissues), was amplified in a final volume of 20 μL, in the presence of ddPCR Supermix for Probes (no dUTP) 2X (Bio-Rad) and TaqMan™ miRNA 20X assays (Thermo Fisher Scientific) (Table 1). 40 μL of droplets emulsion was automatically generated using Automated Droplet Generator (AutoDG) (Bio-Rad) and amplified in a thermal cycler. The following thermal cycler conditions were used: 95 °C for 10 min, 40 cycles of 95 °C for 15 s and 60 °C for 1 min and a final step of 98 °C for 10 min. Droplets were analyzed using the QX200 Droplet Reader, and data analysis was performed with QuantaSoft version 1.7.4 (Bio-Rad).

## Results

### Study workflow: cells and tumor xenografts as models for liquid biopsy

**Fig. 1:**
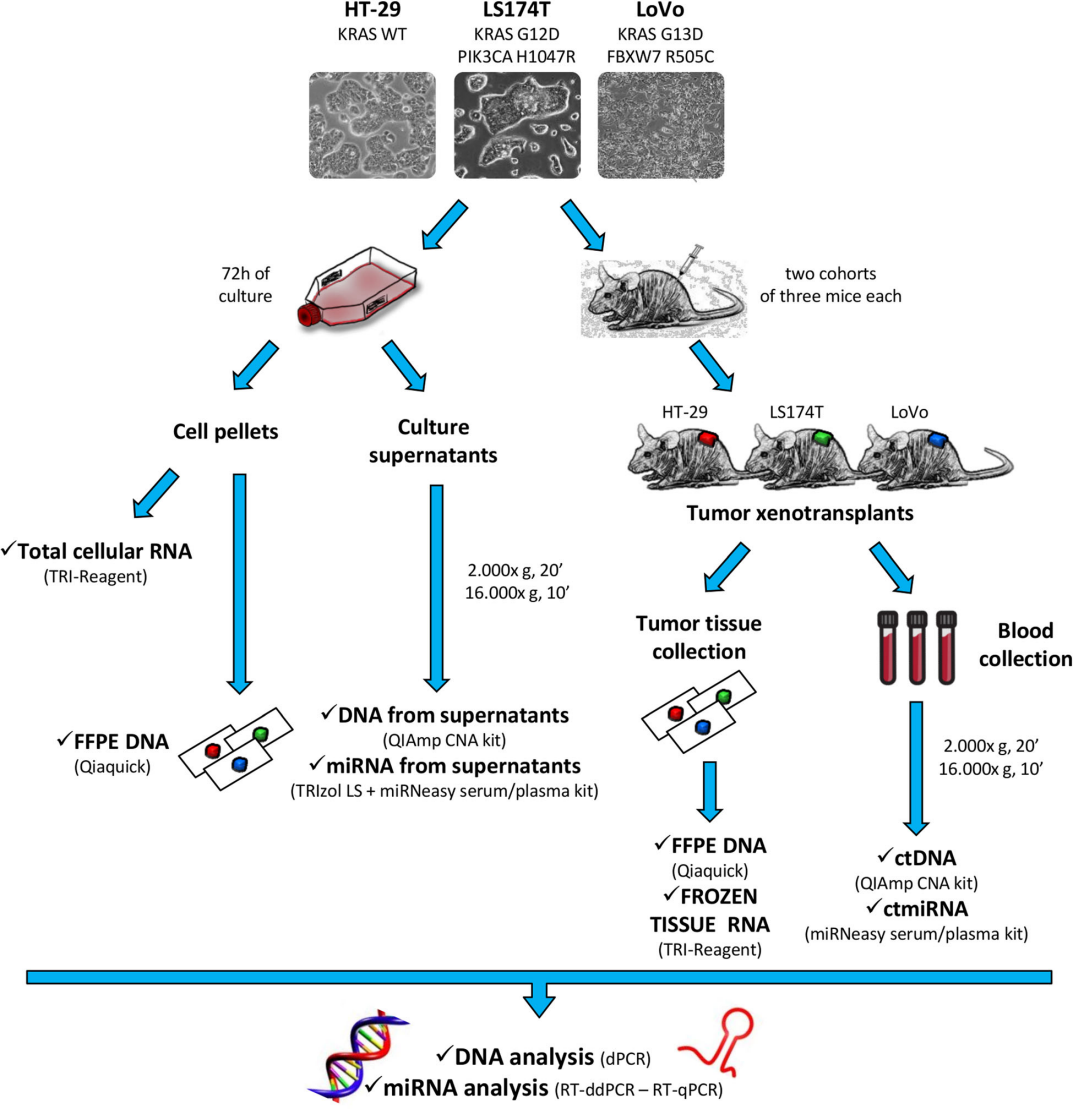
Study workflow. Three human colorectal cancer cell lines (HT-29, LoVo and LS174T) were selected as proxies of clinical cancers and cultured in vitro (left) or used to establish tumor xenografts (right). DNA and RNA were isolated from cells, supernatants and tumor xenografts. ctDNAs and miRNAs were isolated from blood plasma. dPCR, RT-ddPCR and RT-qPCR were performed to detect KRAS G12D and G13D, PIK3CA H1047R, FBXW7 R505C mutations and miR-141, miR-221 and miR-222 expression levels

To assess ctDNAs and miRNAs, a model study was designed that includes cells, culture supernatants, tumor xenografts, and blood samples, as outlined in Fig. 1. Three different CRC cell lines (HT-29, LoVo and LS174T) were selected as proxies of clinically evident cancers and sources of soluble analytes. Because *RAS* mutations are assessed in the routine diagnosis of colorectal carcinoma to assign epidermal growth factor receptor (EGFR) blockade therapy [10], we selected cells that are either wild-type for KRAS (HT-29), or carry the G12D and G13D mutations (LS174T and LoVo, respectively). DNAs and miRNAs were extracted from both cells and culture supernatants, as outlined in Fig. 1 (left). The same cells were also modeled as xenotransplants in nude mice (Fig. 1, right). Matched tumor tissue and blood samples were taken at sacrifice. All biological specimens were then subjected to dPCR, ddPCR and RT-qPCR analysis as outlined in Materials and Methods.

### Genomic and ctDNAs in cells, supernatants, xenotransplants and mouse blood

**Fig. 2:**
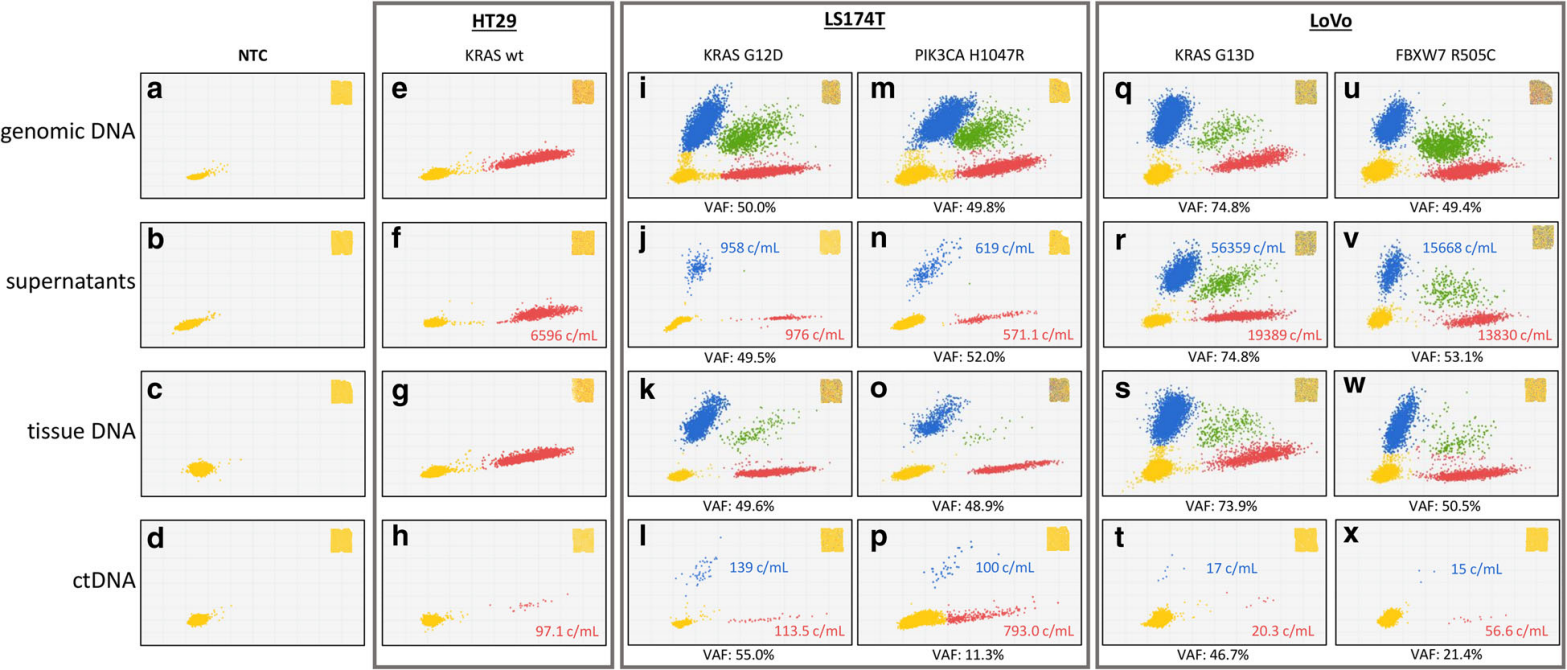
dPCR analysis of KRAS status in cell lines, culture supernatants, tumor tissue xenotransplants and mouse plasma. Input DNAs (from FFPE specimens, culture supernatants and ctDNAs) were subjected to chip-based dPCR using a duplex TaqMan assay that discriminates between WT (red) and mutated KRAS (blue) amplimers at codons 12 and 13, as indicated. Double-positives (microwells containing both WT and mutated KRAS) are shown in green. Microwells in which no PCR amplification takes place are represented by yellow dots (lower left). Variant Allele frequency was calculated by dividing the number of mutated DNA by the total number (mutant +WT) of amplified alleles. Copies per mL are noted and color-coded in supernatants and plasma samples. A whole chip view is depicted in the insets as a quality control

DNAs were obtained from: (1) cell pellets of HT-29, LS174T and LoVo cells grown in culture flasks to approximately (±5%) the same numbers; (2) equal amounts of supernatants from the above flasks; (3) genomic DNAs from tumor xenotransplants grown to 300 mm^3^ (±15%) in size (all tumors); and (4) equal amounts of pooled blood plasma obtained from the above animals (*n* = 6). DNAs and ctDNAs were subjected to dPCR for KRAS G12D and G13D, and for control PIK3CA H1047R and FBXW7 R505C mutations, to test DNA fragments originating from the same (KRAS) as well as distinct (PIK3CA and FBXW7) genomic loci in two different (LS174T and LoVo) CRC cell lines. Mutated *RAS* sequences were detected in LoVo and LS174T (Fig. 2, panels I-X), but not in KRAS-WT HT-29 cells (E-H), as expected. VAFs in cells and tissues were approximately 50% in all cases (I, M, K, O, U, V), as expected, with the exception of KRAS in LoVo (Q and S, approximately 75%), confirming copy number variation of the KRAS MUT allele in the absence of chromosome 12 duplication (see whole genome sequencing data in the *Cancer Cell Line Encyclopedia* at *https://portals.broadinstitute.org/ccle*). However, and interestingly, when free soluble DNAs were considered, a strikingly higher variability was readily observed in both VAFs and absolute copy numbers per mL. Specifically, KRAS G12D DNA from LS174T was almost 60 times less abundant than G13D DNA from LoVo (958 vs 56,359 copies/mL; compare J and R) in culture supernatants and, *viceversa*, it was approximately 8 times more abundant (compare L and T; 139 vs 17 c/mL) in blood, demonstrating a 500-fold difference in the ability of the two cell lines to generate accumulation of RAS ctDNA in cell supernatants vs blood of tumor-xenotransplanted mice. Likewise, comparison of PI3KCA and FBXW7 (compare N to V, and P to X) demonstrated greater (25 times) and lesser (7 times) relative abundances of the two mutations in culture supernatants and blood, resulting in a 175-fold difference that is in full agreement with KRAS ctDNA blood accumulation preferences (LS174T > LoVo). Altogether, ctDNA levels appear to reflect a cell line-dependent property only marginally affected by the genomic regions of origin of the soluble analytes. Preferential ctDNA accumulation in the blood of LS174T xenografts was reproduced in: (a) two separate cohorts of 6 mice carrying larger (1000 mm^3^) tumors, (b) two additional, distinct plasma samples obtained by pooling blood from two cohorts of three animals each, and (c) two plasma samples from two individual animals (data not shown). Altogether, our results suggest the existence of a minimum of two gateways. The first gateway predominantly operates in LS174T cells by controlling DNA scavenging towards the microenvironment, since this cell line releases in vitro much less DNA than LoVo. Conversely, the latter gateway becomes evident in LoVo cells when they are grown as tumor xenografts, and prevents the accumulation of mutated ctDNA in blood.

### Cellular and histopathological features potentially associated with ctDNA accumulation

At least 3 major cellular/histopathological features are known to account for DNA accumulation in the extracellular milieu and/or in blood: cell proliferation, tissue necrosis and tumor micro-vasculature [4]. A simple explanation of ctDNA accumulation based on cell proliferation is unlikely, since LS174T proliferate much faster than LoVo cells both in culture and as xenografts (doubling times of 24 h vs 96 h in culture, and 4 days vs 17 days in the mouse, respectively), but the preferential accumulation of LS174T DNA is evident only in blood. It may then be hypothesized that this preferential ctDNA release might depend on the tissue architecture, e.g. necrosis or micro-vasculature. However, morphological evaluation revealed higher percentages of necrotic areas in low-ctDNA-releasing LoVo as compared to LS174T xenografts (30% vs 45%). Moreover, staining with antibodies to the endothelial marker CD31 demonstrated very similar numbers (about 14/microscopic field) of blood micro-vessels in LS174T and LoVo xenografts (Additional file 1: Figures S1 and S2). These results suggest that ctDNA gateways operate independently of tissue architecture, e.g. they are likely dependent on constraints on ctDNA release that operate both at single-cell and whole-body levels, but independent from housekeeping functions associated with cell proliferation, tumor necrosis and tumor vascularization.

### miRNAs in cell lines and culture supernatants

**Fig. 3:**
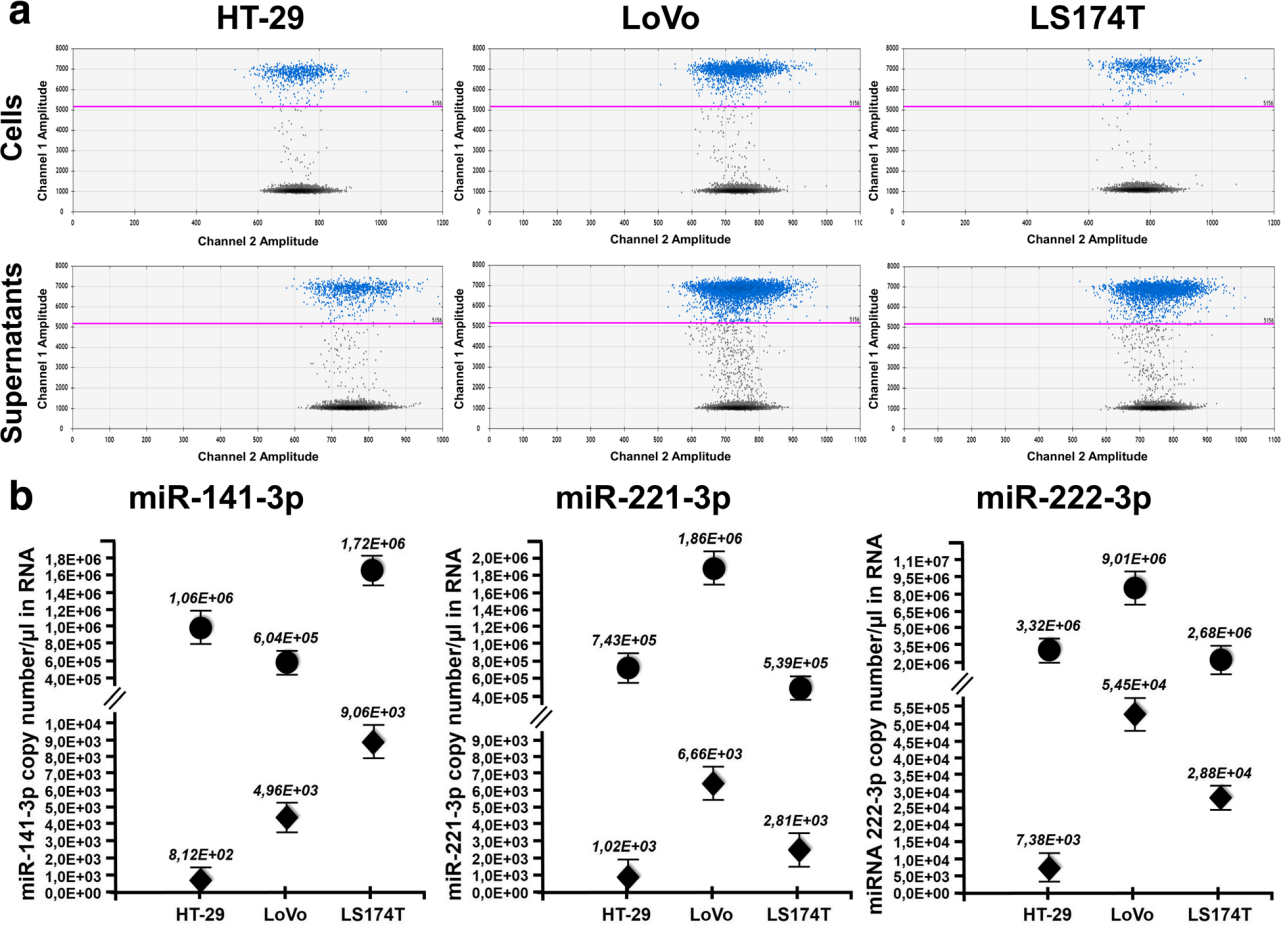
miRNA expression in cultured cells and supernatants. (**a**) 2D ddPCR plots of miR-222-3p in the three indicated cell lines (upper) and their culture supernatants (lower). All cDNAs from cells were diluted 1:100 for accurate Poisson distribution analysis. (**b**) miR-141-3p, miR-221-3p and miR-222-3p were quantitated in cells (dots) and supernatants (diamonds). All data are normalized for total mRNA content, and expressed as copy/μl of miRNA in the original samples. Standard deviation was calculated from three independent experiments

Next, we wondered whether similar gateways regulate circulating miRNAs. Three miRNAs (miR-221, miR-222 and miR-141) were selected since they were previously described to affect colorectal cancer biology and clinical behavior [11, 12]. RNAs from dry frozen pellets and the corresponding culture supernatants were reverse transcribed, and amplified by ddPCR. 2D plots of representative miR-222-3p experiments and the complete dataset for the three miRNAs (cell lines and supernatants) are shown in Fig. 3a (top and bottom panels respectively). In parallel, in Fig. 3b, are reported the copy number/μl of RNA of all the three miRs analized in cells and supernatants (dots and diamonds respectively). From Fig. 3 it may be seen that miR-222 is more abundant in the supernatants from the cells in which it is expressed most (for instance 5.45^4^ and 7.38^3^ miR-222 molecules/μl of RNA are present in the supernatant of LoVo and HT-29 cells, respectively; similarly, the intracellular accumulation of miR-222 was 9.01^6^ and 3.32^6^ molecules/μl of RNA). Data reported in Fig. 3b indicate that miR-221 and miR-222, that belong to the same miRNA cluster, are proportionately expressed in cells and supernatants, whereas miR-141 levels do not correlate between cells and supernatants, since HT-29 cells express high levels of miR-141 (1.06^6^ molecules/μl of RNA, compared the lower 6.04^5^ amounts of LoVo cells) but do not efficiently release it into the medium (8.12^2^ molecules/μl of RNA, compared the higher 4.96^3^ amounts of LoVo supernatants). Additional ddPCR results are displayed in Additional file 1: Figure S3 and were confirmed by RT-qPCR (Additional file 1: Figure S4), although this latter technique systematically overestimated miRNA concentrations. To exclude that bovine miRNAs (that are contained in FBS and are highly homologous to human miRNAs) might contribute significant noise to PCR assays aimed at tumor miRNAs, we reverse transcribed from complete culture medium and from medium conditioned by cells grown in culture to 70% confluency. As shown in Additional file 1: Figure S5, the contribution of bovine miRNA was negligible. Altogether, these results rule out trivial technical artifacts and demonstrate a specific gateway preventing miR-141 release into HT-29 supernatants. When the results shown in Fig. 3 are taken together with those shown in Fig. 2, it can be concluded that, similar to ctDNAs, miR-141 was released into HT-29 cell supernatants 10 and 6.5 times less abundantly with respect to LoVo and LS147T cells, respectively; on the contrary, release of miR-141 in blood of HT-29 xenografted mice was found similar to that observed in the blood of LoVo and LS147T xenografted mice.

### miRNAs in tumor tissues and plasma from xenografted mice

**Fig. 4:**
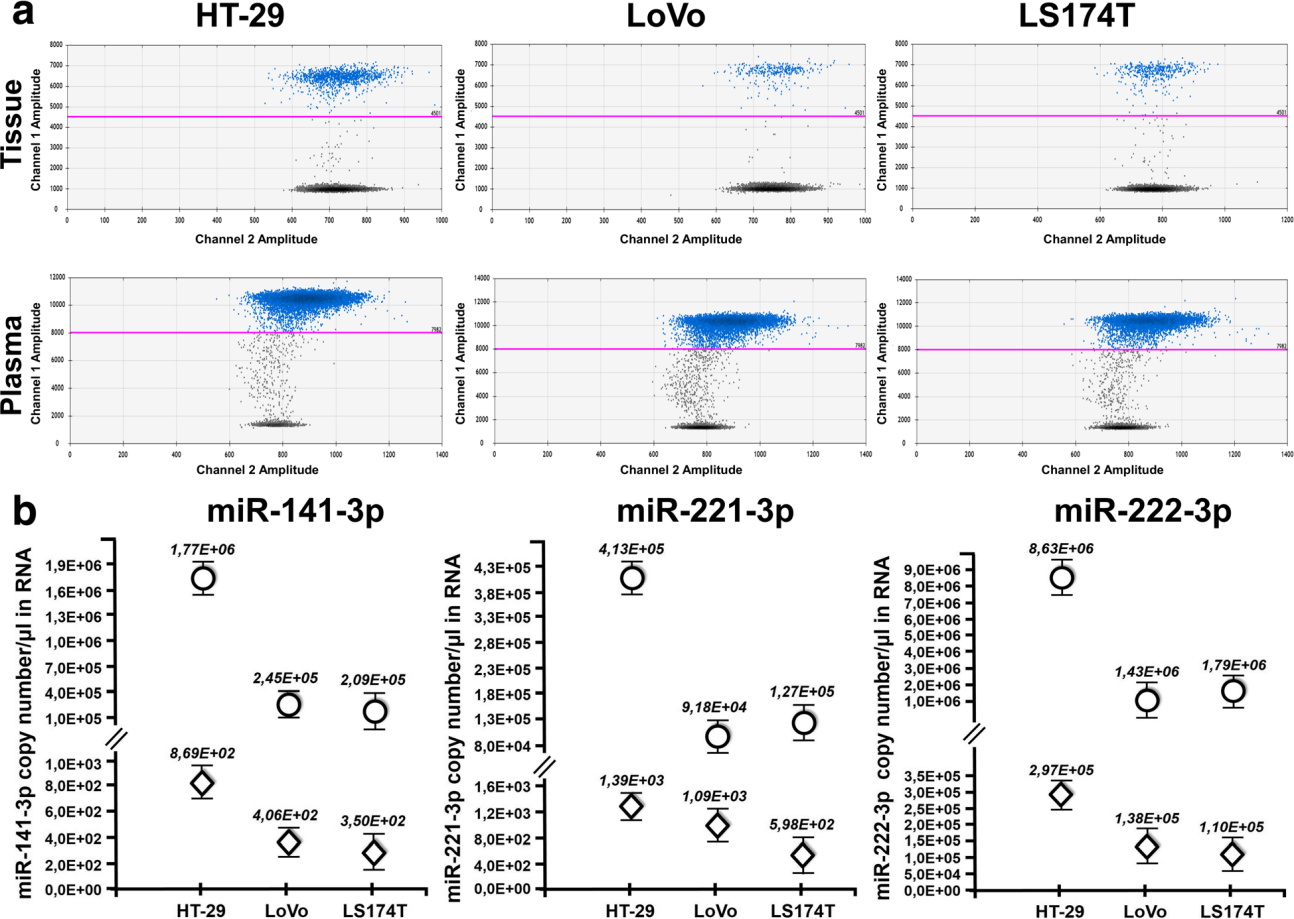
RT-ddPCR analysis of miRNA expression in tumor xenografts and plasma. Tumor tissue and blood plasma were obtained from three mice, and miRNAs were quantitated by RT-ddPCR. (**a**) 2D ddPCR plot of miR-222-3p in tumor tissues (upper) and plasma (lower). 2D ddPCR plots were from 1:100 diluted samples for optimal Poisson distribution. (**b**) ddPCR analysis of miR-141-3p, miR-221-3p and miR-222-3p from tumor tissue (dots) and plasma (diamonds). All data are expressed as copy/μL of miRNA from the original sample. Standard deviation was calculated from three independent experiments

**Fig. 5:**
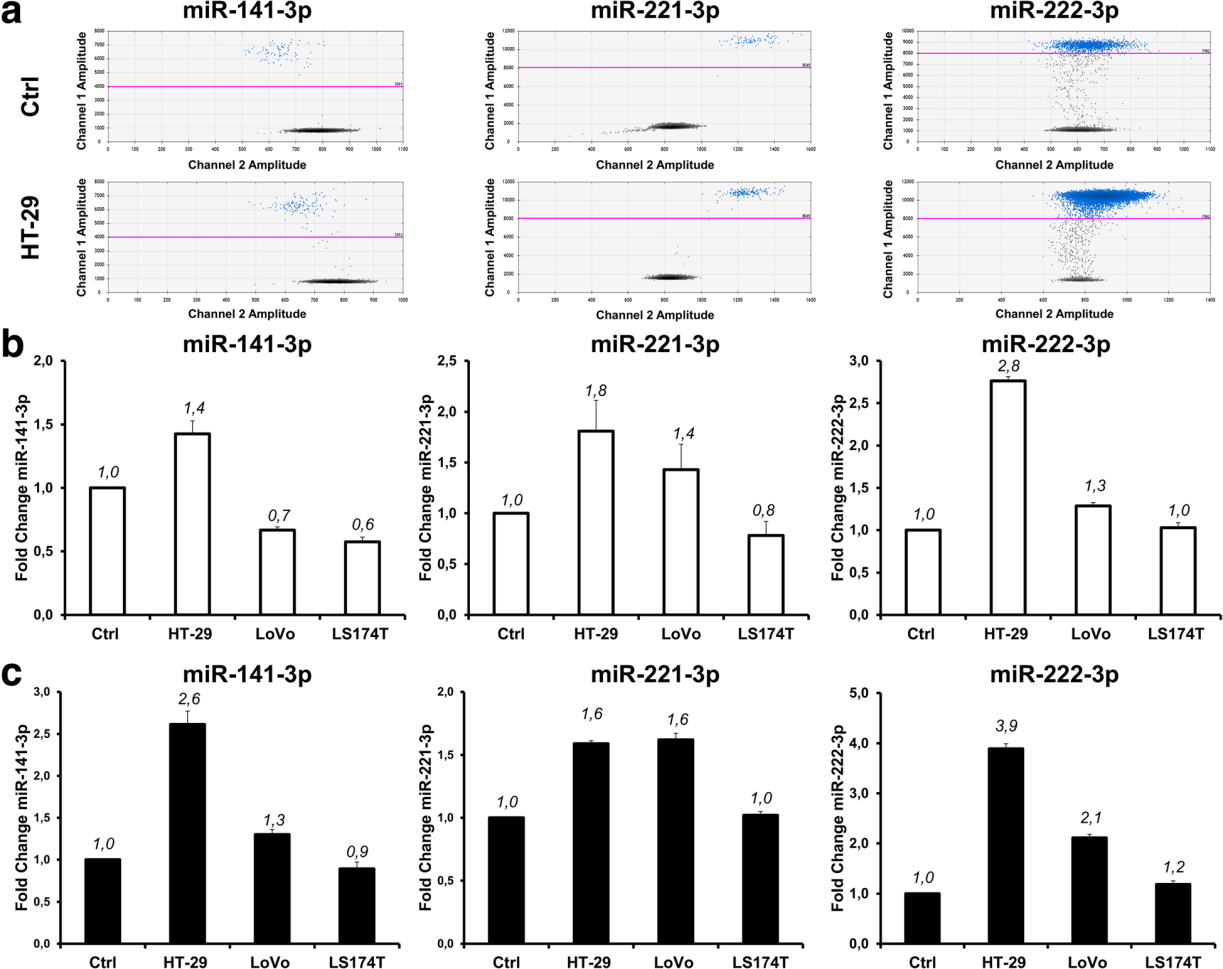
Comparative analysis of miRNA expression in plasma samples of xenotransplanted mice and tumor-free littermates. Plasma samples were analyzed by RT-ddPCR (**a** and **b**) and RT-qPCR (**c**). Panel **a** displays representative 2D ddPCR plots from tumor-free (top) and tumor xenotransplanted (bottom) mice. Panels **b** and **c** show miRNA ratios between xenotransplanted and tumor-free mice for the three miRNAs, as estimated by ddPCR and RT-qPCR, respectively

Next, we analyzed tumor xenotransplants and their corresponding plasma specimens using the same approach (Fig. 4). A representative example of miR-222 content is shown in Fig. 4a and all the quantitative data for miR-141, miR-221 and miR-222 are given in Fig. 4b. This resulted in a graphical outline very similar to that of Fig. 3. Comparison of the two figures (representative 2D ddPCR plots and the complete dataset from all xenotransplants in the top and bottom panels, respectively) demonstrates at glance that miR-141, poorly released in the culture supernatant (Fig. 3), is instead clearly detectable in plasma (Fig. 4) as the other microRNA analyzed, i.e. miR-221 and miR-222. Also in this case, miRNA levels were independently assessed by RT-qPCR, that confirmed ddPCR results (Additional file 1: Figures S6 and S7). Finally, we investigated whether cross-species miRNA homology might influence our in vivo results. To this end, we quantitated baseline, endogenous miR-221, miR-222 and miR-141 levels in tumor-free, healthy nude mice. Both RT-ddPCR and RT-qPCR demonstrated that circulating miRNAs were detectable even in the absence of tumor growth (Fig. 5). Thus, whereas bovine miRNAs do not contribute significant background in vitro (see above), mouse miRNAs do, and this affects the interpretation of the in vivo results. The differences between tumor-bearing and tumor-free mice were clearly appreciable for all the three miRNAs analyzed on HT-29 xenografts. In particular, it should be underlined that, miR-141 was significantly more abundant in the plasma of mice bearing HT-29 tumor xenografts as compared to their tumor-free littermates. This result is compatible with the hypothesis suggesting that tumor masses induce systemic miR-141 accumulation in spite of the intrinsic poor ability of HT-29 cells grown in vitro to release this miRNA in the culture supernatant.

## Discussion

Both circulating tumor DNAs and miRNAs are being increasingly applied as early diagnostic, prognostic and companion biomarkers of target therapy, particularly in a liquid biopsy setting [4]. Liquid biopsy is a powerful tool applicable to all or most human cancers, including colorectal, lung, melanoma, and breast neoplasms, to cite a few, but a detailed knowledge of the molecular mechanisms underlying the release of circulating analytes is still lacking.

Experimental modeling by tumor xenografts has been proposed to characterize the rules of ctDNA [13–16] and miRNA [17–20] release into the bloodstream. In all these studies, ctDNAs and miRNAs were shown to be released into the bloodstream following upstream, ‘default’ or ‘housekeeping’ cellular events. ctDNAs accumulated as a result of apoptotic and non-apoptotic pathways involving DNA fragmentation and DNA packaging with shuttle proteins. miRNAs accumulated as a result of their conventional biogenesis in subcellular vesicles. The above studies demonstrated that analytes release can be successfully modeled in the mouse.

In this report, we wondered whether a substantial analyte fraction might be lost before ctDNAs and miRNAs hit the bloodstream, or in the bloodstream itself, e.g. whether analyte release is finely tuned downstream of default biogenesis. Interestingly, we found that non-housekeeping, cell line-specific ‘gateways’ operate on specific analytes, and may have opposite influences on a given analyte in vitro and in vivo. For instance, we found that DNAs, regardless as to whether they are from the same (KRAS mutations at codons 12/13) or different (PIK3CA and FBXW7 mutations) genomic regions, are all more efficiently released from LoVo than LS174T cells, whereas the reverse occurs when these tumor cells are grown as xenotransplants, e.g. LS174T ctDNAs are much more abundant in blood. Cell line-specific preferences are estimated, depending on the specific DNA, to range from 175 to 500-fold, and do not appear to depend on ‘housekeeping’ factors such as cell proliferation, growth of tumor xenografts, necrosis, or microvasculature density. This implies a minimum of two separate, finely tuned, gateways/checkpoint: (a) the first gateway blocks ctDNA release from cells and is particularly efficient in LS174T; (b) the second gateway is distinct, prevents steady-state accumulation in blood, presumably operates in both xenotransplants, but it is more evident in LoVo. These gateways act at the cell and whole-body levels, respectively, but their precise nature will require further investigation.

Essentially the same concept applies to miRNAs. As to the least abundant (in our set) miR-141, it was released by all tested cell lines much less efficiently than miR-221 or miR-222, and this was particularly evident in HT-29 cells (see the quantitative values of Fig. 3 and Fig. 4). By contrast, miR-141 was clearly detectable in the bloodstream of HT-29 xenotransplanted mice. At first sight, one might interpret these results as a gateway removal in vivo. However, murine miR-141 (identical to its human orthologue) is abundant in tumor-free mice. Thus, from the present data it is impossible to determine whether the cellular release gateway opens up in vivo (e.g. HT-29 cells start releasing miR-141 when grown as tumor xenografts) or miR-141 accumulates as a result of a xenograft-mediated systemic effect (e.g. ctmiR-141 is contributed by normal mouse cells as a result of tumor implantation). Thus, whereas *RAS* mutations can be specifically detected in tumor-bearing mice with no interference of endogenous WT *RAS* murine sequences (Fig. 2), circulating endogenous murine miRNAs indistinguishable from their human counterparts are present in tumor-free mice and may be a confounding factor when interpreting results.

Regardless, our data demonstrate that gateways on the one hand, and/or host systemic effects induced by the tumor on the other, influence the presence of ctDNAs and miRNAs and can be identified. Thus, careful modeling in mice may considerably help (with caveats) in selecting optimal biomarkers for clinical use as well as validate anti-miRNA therapeutic approaches [21–23].

From a more general viewpoint, tumor-xenotransplanted mice and other in vivo models may have an important role because they resolve biological variables from technical variables (such as handling and storage of biological fluids, pre-analytical processing, as well as DNA and RNA isolation protocols) that might affect efficient marker detection by liquid biopsy [24–28]. Finally, tumor-bearing mice may be useful to compare different analytical strategies including, among others, different PCR/RT-PCR and NGS platforms, instruments and protocols, as well as PCR-free methods [29].

## Conclusions

Although the number of ctDNAs and miRNAs taken into account in the present study is limited, our data clearly show in a strictly controlled setting that different cell lines give rise to very different liquid biopsy ctDNA and miRNA profiles. Assimilating each of the three tested cell lines/xenografts to a distinct tumor growing in a different patient, it is tempting to speculate that multiple, finely tuned (non-housekeeping) control gateways exist that selectively regulate the release/accumulation of distinct ctDNA and miRNA species. Tumors from different patients may then considerably differ in gateway usage, adding several layers of complexity to the well-known idea of molecular heterogeneity. For instance, a mutated DNA or miRNA highly represented in the tumor may not be proportionately represented in the blood of a given patient. Analyte under-representation will make it more difficult than expected to interrogate the clinical variety of colorectal tumors. On this basis, it is predicted that very large panels of index aberration are necessary, and that future, careful modeling in mice may considerably help to identify the analytes most affected by individual gateways. Enlarged sets of cell line xenografts and, preferably, patient-derived xenografts are necessary to this end.

## Additional file

Additional file 1: Supplementary analysis. (DOCX 4152 kb)

## Abbreviations

CEA: Carcinoembryonic antigen
CRC: Colorectal carcinoma
ctDNA: Circulating tumor DNA
ctmiRNA: Circulating tumor microRNA
dPCR: Digital PCR
EGFR: Epidermal growth factor receptor
FFPE: Formalin fixed and paraffin embedded
PCR: Polymerase chain reaction
RT-ddPCR: Reverse transcription- droplet digital PCR
RT-qPCR: Reverse transcription quantitative PCR
VAF: Variant Allelic Frequency
