# Supplementary material for: Liquid biopsy in mice bearing colorectal carcinoma xenografts: gateways regulating the levels of circulating tumor DNA (ctDNA) and miRNA (ctmiRNA)
Source: J Exp Clin Cancer Res. 2018 Jun 26;37:124. doi: 10.1186/s13046-018-0788-1 (PMC6020232; doi:10.1186/s13046-018-0788-1)
Supplement: Supplementary file 1 — Supplementary analysis. (DOCX 4152 kb) [file 13046_2018_788_MOESM1_ESM.docx]

**Liquid biopsy in mice bearing colorectal carcinoma xenografts: gateways regulating the levels of circulating tumor DNA (ctDNA) and miRNA (ctmiRNA)**

Jessica Gasparello^1*^, Matteo Allegretti^2*^_,_ Elisa Tremante^2^, Enrica Fabbri^1^, Carla Azzurra Amoreo^3^, Paolo Romania^2^, Elisa Melucci^3^, Katia Messana^2^, Monica Borgatti^1^, Patrizio Giacomini^2#^, Roberto Gambari^1#^ and Alessia Finotti^1^

^1^Department of Life Sciences and Biotechnology, Ferrara University, Ferrara, Italy

^2^Oncogenomics and Epigenetics, IRCSS Regina Elena National Cancer Institute, Rome, Italy

^3^Pathology, IRCSS Regina Elena National Cancer Institute, Rome, Italy

*These authors contributed equally to this work

^#^ Corresponding authors

**ADDITIONAL FILE MATERIALS**

**ADDITIONAL FILE METHODS**

**Technical information on RT-qPCR: standard miRNA curves.**

For absolute quantification using RT-qPCR, a standard curve for each miR was obtained by using synthetic miRNAs (custom-synthesized by Integrated DNA Technologies, Coralville, IA, USA; Table S1) at incremental concentration (from 0.018 to 60 amoles for each well). 3 µL of cDNAs were amplified for 50 cycles using the previous primers and TaqMan Universal PCR Master Mix, no AmpErase UNG 2x (Thermo Fischer Scientific) in a CFX96 Touch Real Time PCR Detection System (Bio-Rad, Hercules, CA, USA). All reactions, including no-template control, were performed in duplicate. Data analysis was performed using Bio-Rad CFX Manager Software version 3.1.

**Histopathology and Immunohistochemistry**

Necrosis was evaluated on conventional Hematoxylin/Eosin tissue slides, scoring the percentage of necrotic areas in the total lesion. Microvessel density was evaluated by staining endothelial cells using an optimal pre-determined dilution (1:10) of a rat monoclonal antibody to CD31 (clone SZ31, Dianova GmbH, Germany), using the ULTRATEK HRP kit (Scy Tek Laboratories, Utah, USA). CD31+ vessels were enumerated in six high-power microscopic fields (HPF, 400x magnification) per section, using a light microscope equipped with an image capture software (DM2000 LED, Leica), and averaged.

**ADDITIONAL FILE RESULTS**

**Histopathology and Immunohistochemistry**

Hematoxylin/eosin stains of tissue sections from CRC xenografs were assessed for necrosis, the extent of which was approximately 30% and 35% in LS174T lesions from smaller (300 mm^3^) and larger (1000 mm^3^) tumors, respectively. LoVo xenografts consistently showed 45% necrosis regardless of tumor size (Fig. S1). CD31 staining demonstrated very similar numbers of blood micro-vessels in tumors of 300 and 1000 mm^3^ (14.0±3.8 and 14.2±5.1, non-significant upon *t test*) per microscopic field in LoVo and LS174T, respectively (Fig. S2).

**miRNA quantification by droplet digital PCR (ddPCR) and RT-qPCR in cells and supernatants.**

2D ddPCR plots and RT-qPCR data are shown for all tested miRNAs (Fig. S3 and S4, respectively). The entire set of data is shown to complete the information provided in Fig. 3.

**miRNAs levels in FBS**

Figure S5 displays a miRNA homology analysis between Bos Taurus (bta) and Homo Sapiens (hsa) (panel A), and the levels of miR-141-3p, miR-221-3p and miR-222-3p in FBS, as assessed by ddPCR and RT-qPCR, respectively (B and C). miRNA content in FBS is about 35 (miR-221), 160 (miR-222) and 47000 (miR-141) times lower than in culture supernatants.

**Comparison between ddPCR and RT-qPCR**.

Figures S4 and S5 show results obtained with ddPCR (Fig. S4) and RT-qPCR (Fig. S5) using RNAs isolated from cultured cells and supernatants as templates. Figures S6 and S7 show results obtained with ddPCR (Fig. S6) and RT-qPCR (Fig. S7) using RNA isolated from tumor tissue and plasma isolated from mice xenotrasplanted with the HT-29, LS174T and LoVo human colorectal cancer cell lines. Overall, these data demonstrate that both ddPCR and RT-qPCR, despite having different sensitivity, generate very similar results, leading to identical conclusions.

**miRNA quantification by droplet digital PCR (ddPCR) and RT-qPCR in xenotransplants and blood.**

2D ddPCR plots and RT-qPCR data are shown for all tested miRNAs (Figs. S5 and S7, respectively). The entire set of data is shown to complete the information provided in Fig. 4.

**ADDITIONAL FILE FIGURES**

**
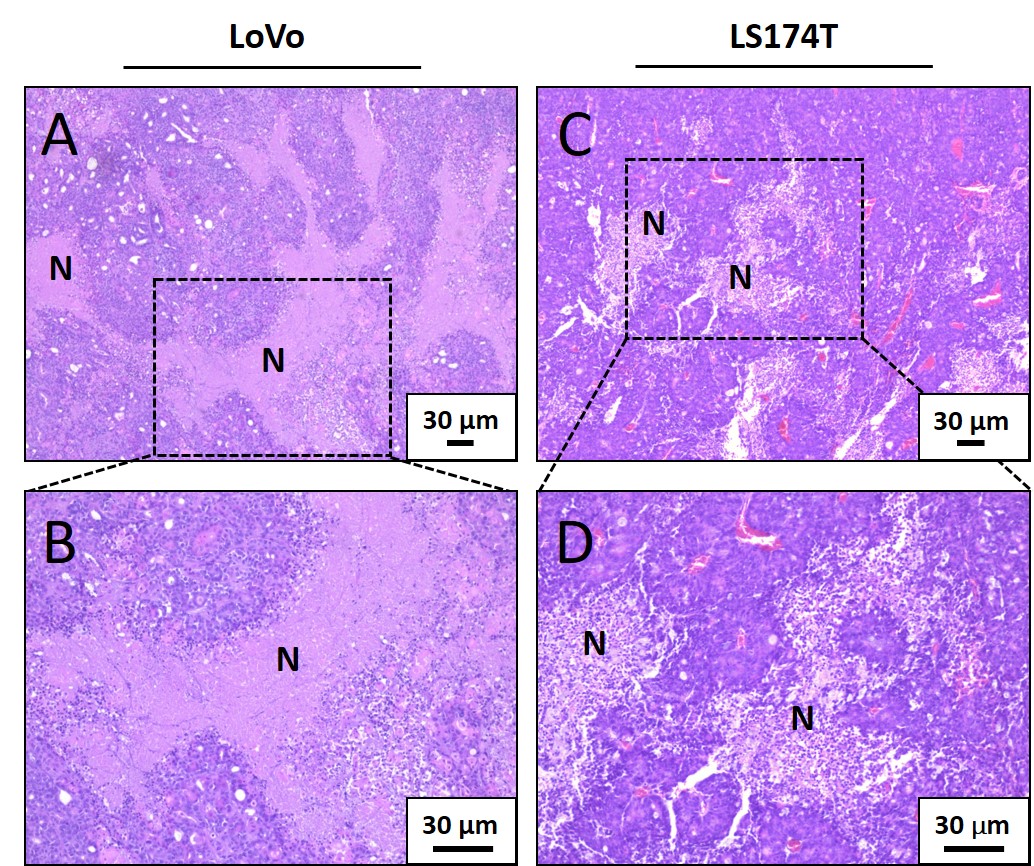
**

**Figure S1. Evaluation of tissue necrosis in tumor xenografts.** Hematoxylin/Eosin low power magnification staining (A and C: 50x; B and D: 100x) of tumor tissue sections from the indicated xenografts (300 mm^3^ in size) were evaluated morphologically. Necrotic areas are noted (N).


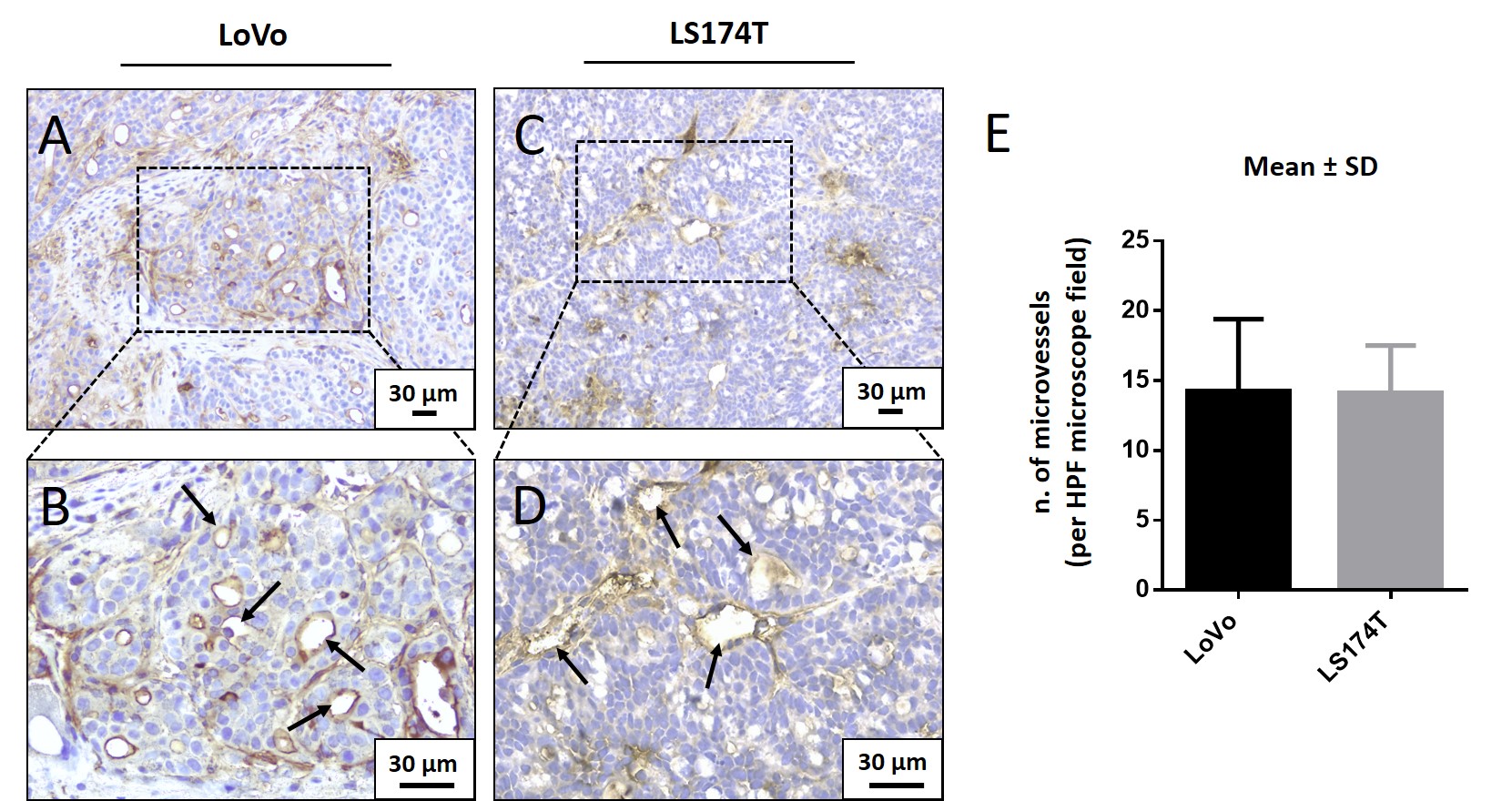


**Figure S2. CD31 immunohistochemical staining of mouse xenotransplants.** Tissue slides of tumor tissue sections from the indicated xenografts grown to 300 mm^3^ in size were stained for the microvessel marker CD31 (arrows in the inset at higher magnification). A and B) 200x magnification. C and D) 400x magnification. The numbers of vessels per High Power Field (HPF) were counted in 2 sets of 3 small (300 mm^3^) and 3 large (1000 mmm^3^) LS174T and LoVo xenografts. E) mean values and standard deviations were averaged (± SD) from all determinations. CD31 counts did not differ in tumors of different sizes.

**
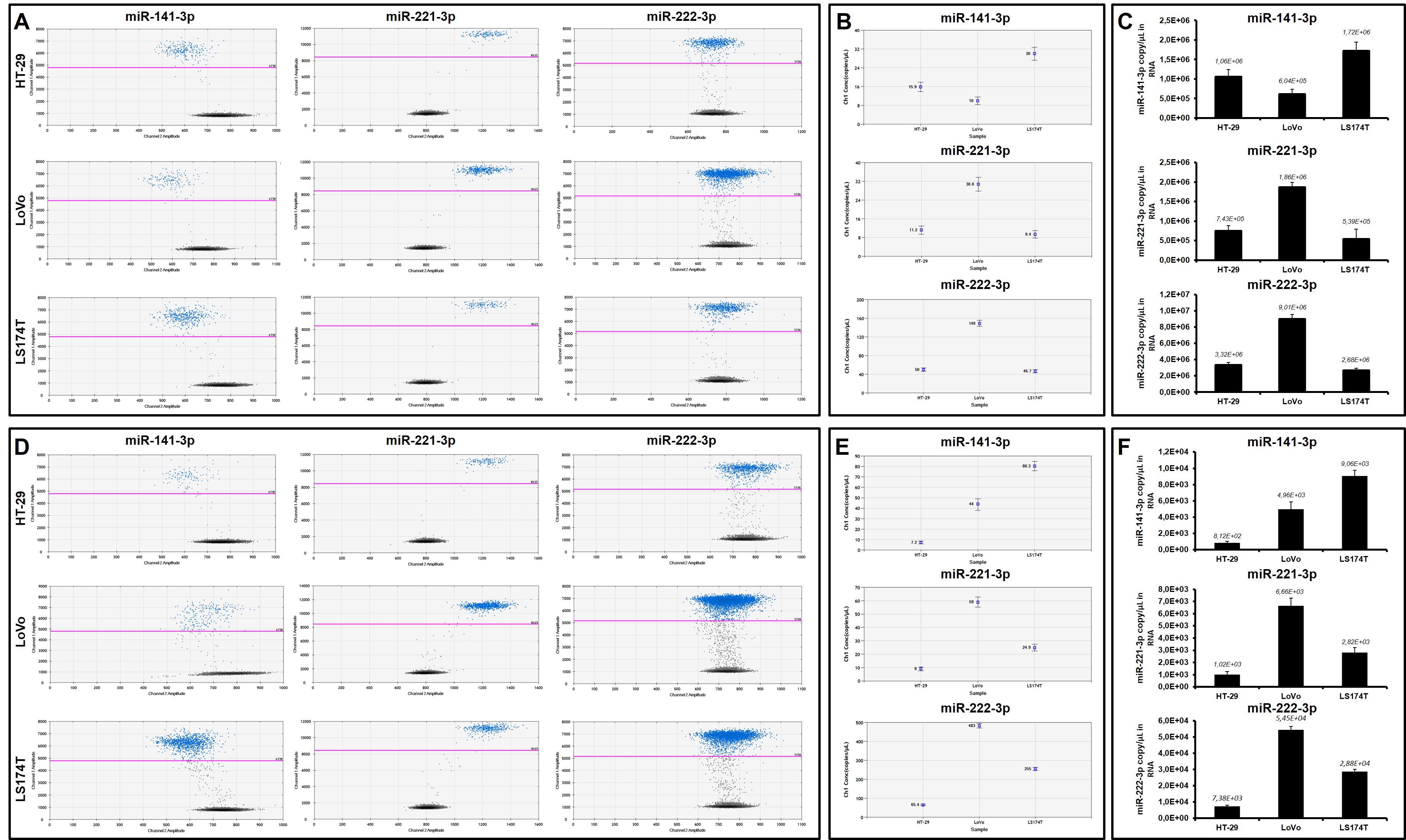
**

**Figure S3. ddPCR analysis of miRNA expression in cultured cells and supernatants.** Before ddPCR analysis, cDNA was diluted 1:100, whereas cDNA from supernatants was used undiluted. miR-141-3p, miR-221-3p and miR-222-3p levels were quantified by ddPCR in both cultured cells (panels A and B) and supernatants (D and E). Data are normalized as copy/µl (C and F). Standard deviation was calculated from three independent experiments.

**
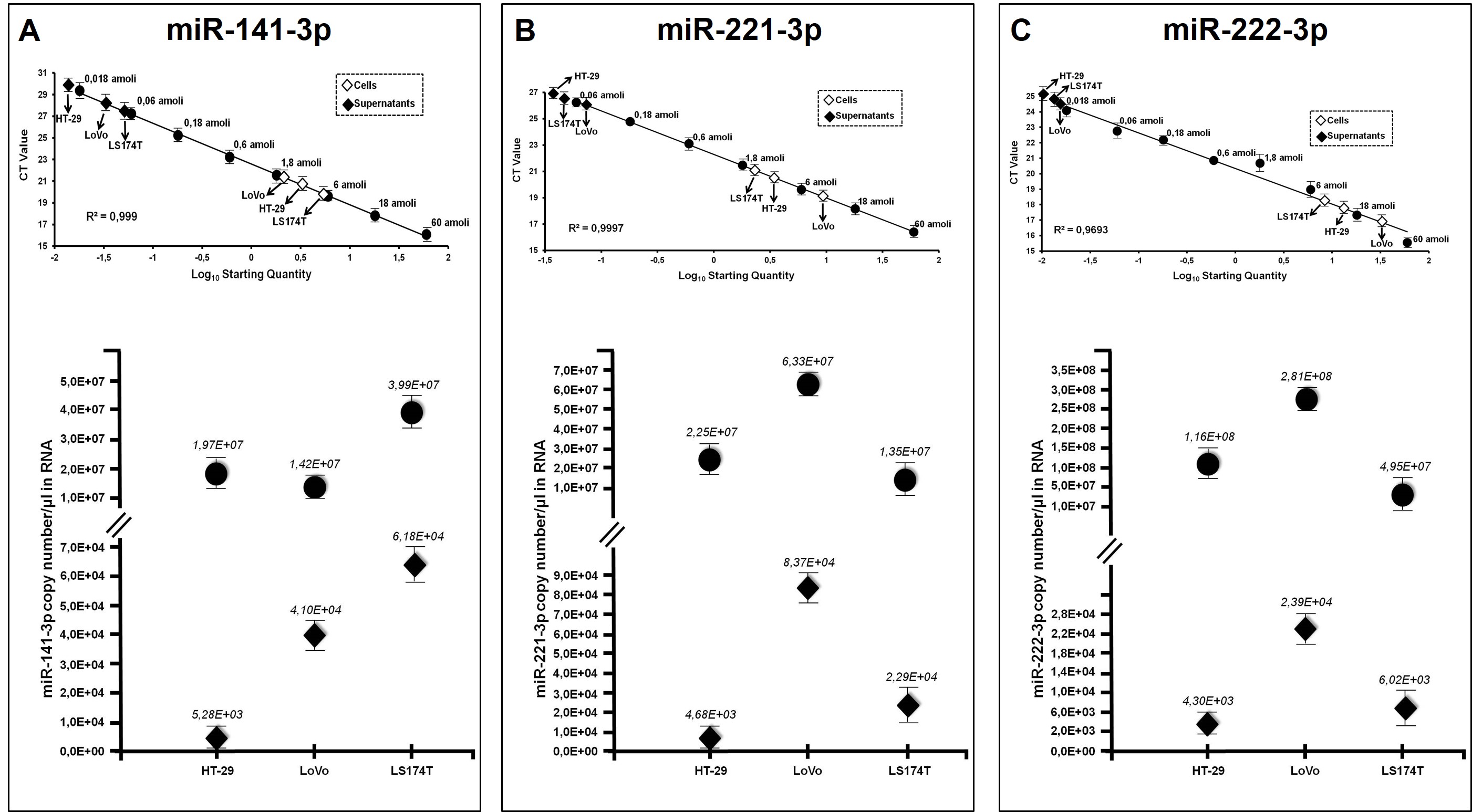
**

**Figure S4. miRNA quantification in cultured cells and supernatants by RT-qPCR.** miR-141-3p (A), miR-221-3p (B) and miR-222-3p (C) expression was quantified by RT-qPCR. A standard curve for each miRNA was obtained using incremental concentration of a synthetic miRNA, and was used as a standard to quantify the absolute concentration of each miRNA in cultured cells (black dots) and in supernatants (black diamonds). All the data are reported as copy/µL. Standard deviation was calculated from three independent experiments.

**
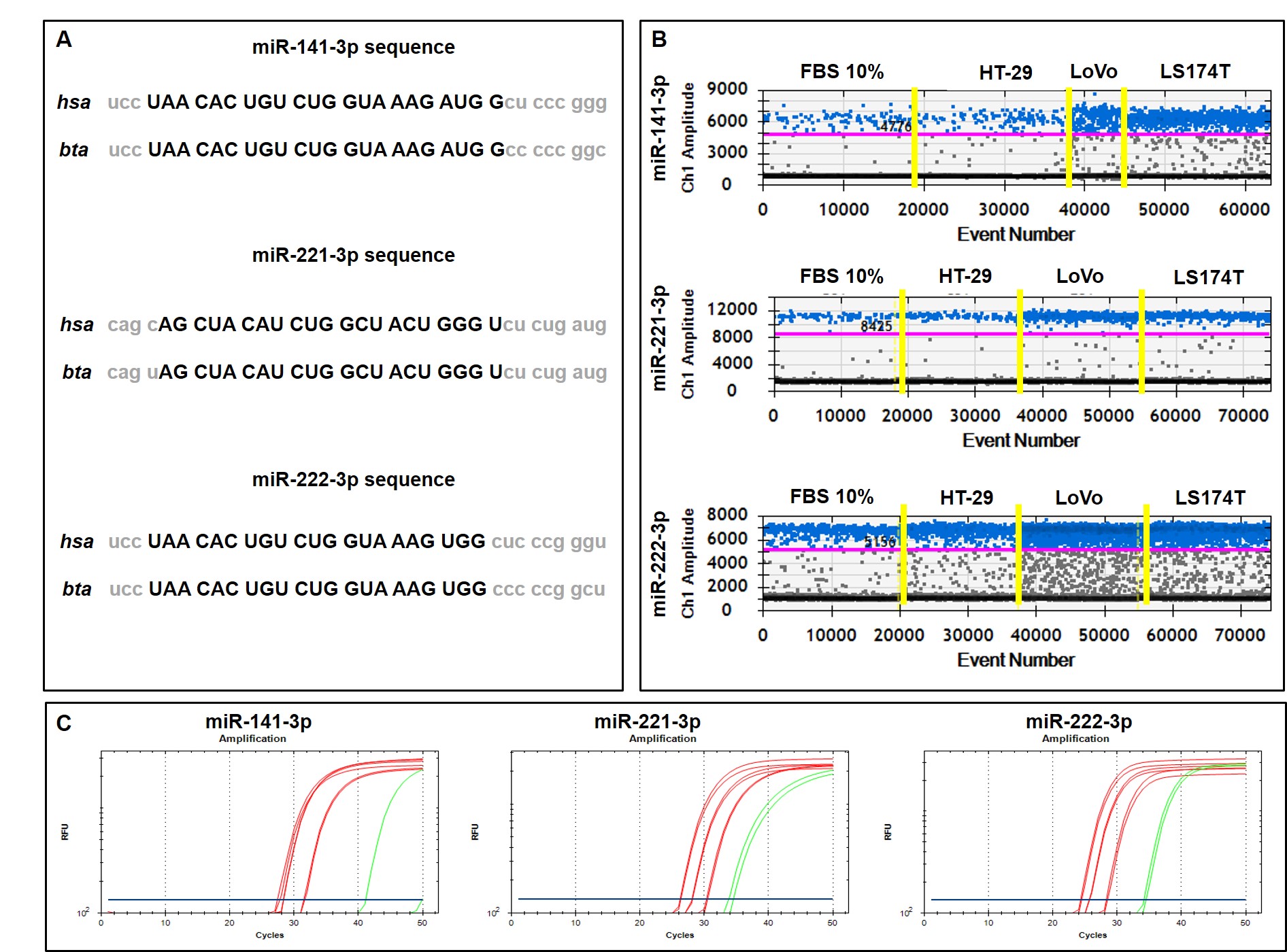
**

**Figure S5. FBS miRNAs levels are very low.** (A) The homology between Bos Taurus (bta) and Homo Sapiens (hsa) miRNAs was analyzed using miRNAminer (groups.csail.mit.edu/pag/mirnaminer). (B, C) miR-141-3p, miR-221-3p and miR-222-3p expression was assessed by ddPCR (plots) and RT-qPCR (curves) in culture supernatants and in RPMI supplemented with 10% FBS (RPMI/FBS). Blue dots are positive events. Grey dots represent negative events. Red curves: cells supernatants. Green curves: RPMI/FBS.

**
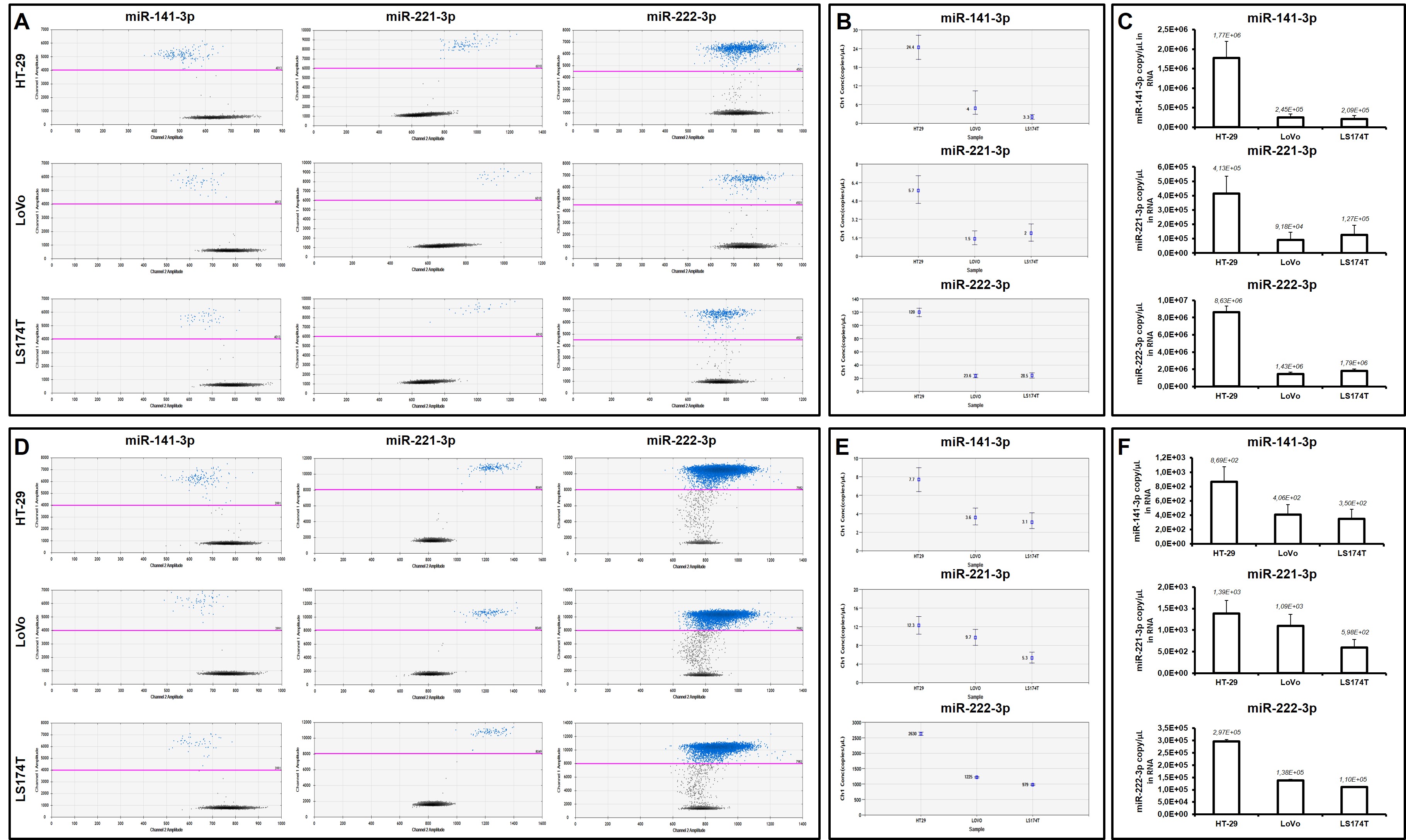
**

**Figure S6.** **ddPCR analysis of miRNAs in tumor xenotransplants and plasma samples.** Before ddPCR analysis, cDNA from tumors was diluted 1:100, whereas cDNA from plasma was used undiluted. (A and D) 2D ddPCR plots showing miRNA expression in tumor tissue and mouse plasma, respectively. miRNA concentrations in ddPCR reaction for tumor tissues (panel B and for plasma samples (panel E) are reported. Data are normalized also as copy/µl in RNA samples (C and F). Standard deviation was calculated from three independent experiments.

**
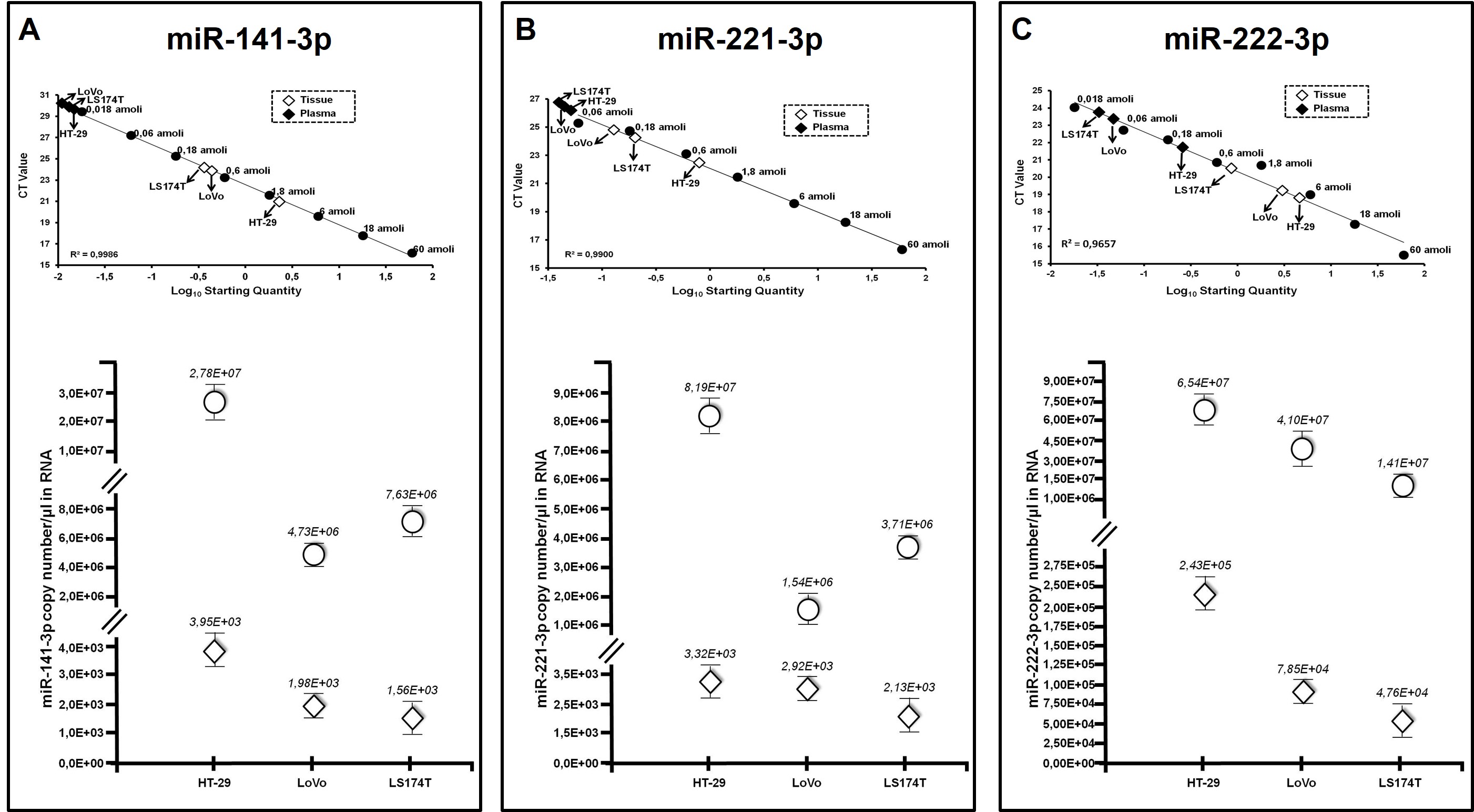
**

**Figure S7.** **miRNA quantification in tumor tissue and plasma by RT-qPCR**. miR-141-3p (A), miR-221-3p (B) and miR-222-3p (C) expression was quantified by RT-qPCR. A standard curve for each miRNA was obtained using incremental concentration of a synthetic miRNA, and was used to quantify the absolute concentration of each miRNA in xenotransplants (open dots) and in plasma (open diamonds). All the data are reported as copy/µL. Standard deviation was calculated from three independent experiments.

**ADDITIONAL FILE TABLES**

**Table S1**. Sequences of synthetic microRNA used for qPCR standard curve generation.

| **microRNA** | **Sequence** | **miRBase Access number** |
| --- | --- | --- |
| hsa-miR-141-3p | 5’- rUrArA rCrArC rUrGrU rCrUrG rGrUrA rArArG rArUrG rG -3’ | MIMAT0000432 |
| hsa-miR-221-3p | 5’- rArGrC rUrArC rArUrU rGrUrC rUrGrC rUrGrG rGrUrU rUC -3’ | MIMAT0000278 |
| hsa-miR-222-3p | 5’- rArGrC rUrArC rArUrC rUrGrG rCrUrA rCrUrG rGrGrU -3’ | MIMAT0000279 |

^a^http://www.mirbase.org/
